# Quantitative ultrasonography for pneumonia

**DOI:** 10.1186/cc14305

**Published:** 2015-03-16

**Authors:** F Corradi, C Brusasco, T Manca, F Nicosia, A Vezzani, V Brusasco

**Affiliations:** 1 Ente Ospedaliero Ospedali Galliera Genova, Italy; 2 Università degli Studi di Genova, Italy; 3 Azienda Ospedaliero-Universitaria, Parma, Italy

## Introduction

Chest-X-ray is recommended for routine use in patients with suspected pneumonia, but its use in emergency settings is limited. In this study, the diagnostic performance of a new method for quantitative analysis of lung ultrasonography was compared with bedside chest X-ray and visual lung ultrasonography for detection of community-acquired pneumonia, using thoracic computed tomography as a gold standard.

## Methods

Thirty-two spontaneously breathing patients with suspected community-acquired pneumonia undergoing computed tomography examination were consecutively enrolled. Each hemi-thorax was evaluated for the presence or absence of abnormalities by chest X-ray and quantitative or visual ultrasonography.

## Results

Quantitative ultrasonography showed higher sensitivity (93%), specificity (95%), and diagnostic accuracy (94%) than chest X-ray (64%, 80%, and 69%, respectively), or visual ultrasonography (68%, 95%, and 77%, respectively), or their combination (77%, 75%, and 77%, respectively).

## Conclusion

Quantitative lung ultrasonography was considerably more accurate than either chest X-ray or visual ultrasonography in the diagnosis of community-acquired pneumonia and it may represent a useful first-line approach for confirmation of clinical diagnosis in emergency settings.
